# Remote Ischemic Preconditioning Enhances Skin Flap Survival via ZNF667/SDF1-Mediated Endothelial Progenitor Cells Functions for Angiogenesis

**DOI:** 10.1007/s13770-026-00817-1

**Published:** 2026-06-06

**Authors:** Yuanbin Li, Jingzhang Li, Zhonglei Liang, Xin Chen, Wei Xiong, Yuhui Huang, Aizhu Qiu, Zhuang Chen, Jianjun Kuang

**Affiliations:** 1 Department of Basic Medicine, Hunan Traditional Chinese Medical College, Zhuzhou, 412012 Hunan China; 2 Department of Thoracic Surgery, Xiangtan Chinese Medicine Hospital, Xiangtan, 411100 Hunan China; 3 Institute of Clinical Pharmacology, Hunan Academy of Traditional Chinese Medicine, Changsha, 410000 Hunan China

**Keywords:** RIPC, ZNF667, EPCs, Flap transplantation, Angiogenesis

## Abstract

*****BACKGROUND***:**

Flap transplantation plays a vital role in wound reconstruction. However, the mechanisms by which remote ischemic preconditioning (RIPC) may improve flap survival remain incompletely understood.

*****METHODS***:**

Rats were randomly assigned to three groups: sham, ischemia/reperfusion (I/R), and RIPC + I/R. The I/R model was established by ligating the iliopsoas and thoracodorsal arteries to induce flap ischemia, followed by reperfusion. RIPC was performed via limb clamping. A combination of high-throughput sequencing, functional cellular assays, and live imaging was used to assess gene expression, cellular functions, and flap viability.

*****RESULTS***:**

RIPC upregulated the expression of ZNF667. This protein acted as a transcriptional repressor of *VHL* by binding to its promoter region, where it competitively inhibited the recruitment of histone-modifying enzymes, including MLL3/4, SETD1A, and EP300. Consequently, histone methylation and acetylation were reduced, leading to suppressed *VHL* transcription. The downregulation of VHL diminished the ubiquitination-mediated degradation of hypoxia-inducible factor-1α (HIF-1α), which in turn enhanced the expression of stromal cell-derived factor 1 (SDF1). This signaling cascade promoted the proliferation, migration, differentiation, and tube-forming capacity of endothelial progenitor cells (EPCs). Live imaging confirmed that RIPC stimulated the recruitment of EPCs into the flap tissue, accompanied by increased microvessel density. These effects collectively enhanced angiogenesis and significantly reduced the area of flap necrosis.

*****CONCLUSION***:**

RIPC improves flap survival by modulating the ZNF667–VHL–SDF1 axis and augmenting the function of EPCs. These findings not only provide a potential therapeutic strategy for flap transplantation but also advance our understanding of the mechanisms underlying flap survival.

**Supplementary Information:**

The online version contains supplementary material available at 10.1007/s13770-026-00817-1.

## Introduction

Flap transplantation is a crucial method for repairing and reconstructing various tissue defects and effectively addresses complex, highly destructive injuries. It plays a vital role in wound healing, particularly when conventional methods are inadequate, such as in complex or chronic wounds [1]. A flap—composed of skin, subcutaneous fat, and occasionally muscle—is surgically transferred from a donor to a recipient site to provide coverage and promote healing. Recent studies have shown that flaps not only offer physical coverage but also contribute to wound healing through mechanisms such as vascularization, immune modulation, and the release of growth factors [1, 2]. A key advantage of using flaps is their ability to enhance local blood supply. Their vascularized nature ensures that the recipient site receives sufficient oxygen and nutrients, which are essential for cellular metabolism and tissue repair [3]. Growth factors released from the flap tissue, including vascular endothelial growth factor (VEGF), stromal cell–derived factor 1 (SDF1), and fibroblast growth factor (FGF), play critical roles in stimulating cell proliferation and migration, thereby accelerating the healing process [4].

Ischemia–reperfusion injury (IRI) is a pathophysiological phenomenon that occurs in organ transplantation, including flap transplantation, and is a major cause of various adverse outcomes. Briefly, IRI refers to the secondary damage that occurs when blood supply is restored after a prolonged period of ischemia, leading to increased local inflammation and the production of reactive oxygen species. The mechanisms of cellular damage involved in IRI include apoptosis, autophagy, necrosis, and necroptosis [5, 6]. The occurrence of IRI is a risk factor for flap failure; if not effectively managed, the large numbers of pro-oxidative free radicals and inflammatory factors generated during IRI can significantly affect flap angiogenesis, ultimately resulting in flap failure [7].

In response, many scholars have explored various strategies targeting IRI based on different mechanisms, aiming to improve the prognosis of flap transplantation. One important strategy involves pharmacological interventions (such as melatonin and heparin) to counteract IRI [8, 9]; however, as of now, no drugs have entered clinical application. Another significant approach focuses on preconditioning the flap, such as ischemic preconditioning (IP). Remote ischemic preconditioning (RIPC) is a type of preconditioning strategy within the category of ischemic preconditioning (IP), distinct from traditional local IP (classic local ischemic preconditioning) [10, 11]. Compared to local IP (such as pre-clamping the flap pedicle), RIPC offers better clinical practicality. As a non-invasive intervention, it can be performed simply by using a limb tourniquet to induce IP, which helps reduce surgical time and eliminates the need for secondary surgeries required for local IP [12]. Currently, randomized controlled trials have confirmed the effectiveness and safety of RIPC [10, 11, 13]; however, its mechanisms of action are not yet fully understood. Clearly, addressing these questions depends on in-depth research into the specific mechanisms of RIPC to further optimize its therapeutic effects.

Angiogenesis in the flap area is crucial for the success of flap transplantation. Endothelial progenitor cells (EPCs) play a critical role in angiogenesis after flap surgery, which is essential for the survival and functionality of the transplanted tissue [14, 15]. These cells, originating from the bone marrow, contribute to the formation of new blood vessels, thereby facilitating the healing process in the flap area. After flap surgery, ischemic conditions often trigger the mobilization of EPCs from the bone marrow into the bloodstream. This process is typically driven by factors such as SDF-1 and VEGF. Enhancing EPC mobilization significantly improves blood flow and tissue viability in flap models, indicating that effective recruitment of these cells is crucial for successful flap healing [16]. Once recruited to the flap area, EPCs differentiate into mature endothelial cells and integrate into the existing vascular architecture. This integration is vital for establishing a functional blood supply to the flap. Given their role in promoting angiogenesis, EPCs have been explored as a therapeutic option to enhance flap survival and healing. Understanding the specific mechanisms by which EPCs contribute to vascular regeneration in flap surgery may lead to improved therapeutic strategies for enhancing flap viability and patient outcomes.

## Materials and methods

### Animal studies

Sprague–Dawley rats [Male (n = 18), aged 6–8 weeks, weighing approximately 200–250 g], were used for the study. All procedures were approved by the Ethics Committee of Hunan Academy of Traditional Chinese Medicine (Approved number: LL20231105) and were conducted in accordance with the National Institutes of Health guidelines for the care and use of laboratory animals. Sprague–Dawley rats were randomly divided into six groups: sham, ischemia/reperfusion(I/R) and RIPC + I/R, RIPC + I/R + ZNF667(KD), RIPC + I/R + SDF1(KD) and RIPC + I/R + AMD3100 groups, with 6 rats in each group. The method of anesthesia remained consistent across all experimental groups. Animals were anesthetized with intraperitoneal injection of ketamine (75 mg/kg) before rat flap surgery. Core body temperature was maintained at 37.0 ± 0.5 °C using a heating pad with a feedback-controlled temperature probe. For flap surgery, the dorsal area of the rat was shaved. A rectangular skin flap measuring 5 cm × 7 cm was marked on the dorsal surface of the rat, with the long axis oriented along the midline. On the left and right sides of their backs, the incision was made from the iliopsoas artery to the thoracodorsal artery, exposing the posterior intercostal artery and separating the subcutaneous tissue from the superficial surface of the deep fascia. At the same time, the intercostal artery was cut, but the iliopsoas artery and thoracodorsal artery were not severed. The elevated flap was sutured in place using 4-0 silk sutures. After the surgery, the rats were placed in a recovery chamber until they regained consciousness. They were monitored for pain and distress and received analgesics (buprenorphine, 0.05 mg/kg) as needed. Animals were excluded if they exhibited signs of severe distress, prolonged hypotension (mean arterial pressure < 50 mmHg), or anesthetic complications (e.g., respiratory depression). The mortality rate during the study period was 5%, primarily due to anesthetic overdose or surgical complications unrelated to RIPC.

The core surgical procedure for flap elevation was identical for all groups. The key difference between groups was the specific intervention applied during this standard procedure:

Sham Group: Underwent the standard flap elevation surgery without vessel ligation.

I/R Group: Underwent the standard flap elevation surgery, with the additional step of ligating the iliopsoas and thoracodorsal arteries for 6h to induce ischemia, followed by reperfusion.

RIPC + I/R Group: Prior to the standard flap elevation and I/R procedure, these rats first received the RIPC intervention. RIPC was induced by three cycles of alternating limb ischemia (5 min of occlusion followed by 5 min of reperfusion). A pneumatic cuff was placed around each of the four limbs and inflated to a pressure of 200 mmHg, which exceeds systolic pressure to ensure complete arterial occlusion.

RIPC + I/R + KD Groups: Lentiviral particles containing short hairpin RNA (shRNA) constructs specifically targeting ZNF667 or SDF1 were injected into the flap area 48h prior to the standard flap elevation and I/R procedure.

RIPC + I/R + AMD3100 Group: Rats received an intravenous injection of the CXCR4 antagonist AMD3100 (1.25 mg/kg in saline) via the tail vein. The injection was administered 10 min prior to the standard flap elevation and I/R procedure. This systemic administration aimed to block the CXCR4 receptor on EPCs, thereby interfering with their recruitment and function.

Flap viability was assessed postoperatively at 7 and 14 days by measuring the area of necrosis and evaluating the color and texture of the flap.

### High-throughput sequencing of skin tissues

Skin samples from the edge of the wound of flap were collected to extract total RNA. The total RNA was sent to Aksomics Life Science & Technology Co., Ltd. (Shanghai, China) for high-throughput sequencing. Briefly, cDNA libraries were prepared from the extracted RNA using a stranded RNA-seq library preparation kit (Thermo Fisher Scientific, Shanghai). The prepared libraries were quantified and normalized before being pooled. High-throughput sequencing was performed on NovaSeq Illumina platform.

### Cell treatments

Rat primary dermal fibroblasts (RDFs) were purchased from Procell Life Science&Technology Co., Ltd. (Wuhan, China). RDFs were maintained in Dulbecco’s Modified Eagle Medium (DMEM, Gibco, Thermo Fisher Scientific) supplemented with 10% fetal bovine serum (FBS, Gibco) and penicillin/streptomycin solution in a humidified incubator with 5% CO_2_ at 37 °C. EPCs were isolated from the peripheral blood of rats. Briefly, mononuclear cells were isolated from the blood by using density gradient medium (Ficoll-Paque). Mononuclear cells were seeded onto six-well plates precoated with fibronectin (5 mg/mL; Corning) in endothelial growth medium (EGM)-2 (Lonza). After 24h in culture, non-adherent cells were removed; adherent cells were continuously cultured in fresh EGM-2 media (Lonza). The EPC phenotype of the isolated cells was confirmed by detection of CD133 and CD34 expession using Flow Cytometry. In addition, the cells were characterized by the uptake of DiI acetylated low-density lipoprotein (DiI-acLDL) and FITC ulexeuropaeus agglutinin-1 (FITC-UEA-1). To induce hypoxia, cells are placed in a hypoxic chamber with low oxygen concentration (1% O_2_) for a predetermined duration (6, 12 or 24h). After the hypoxic treatment, cells are returned to normoxic conditions to induce the hypoxia/reoxygenation (H/R) damage.

### Transfection

Expression vectors of ZNF667, NCOA1 and VHL were constructed by GenePharma Life Science & Technology Co., Ltd. (Shanghai, China). The expression vector is under the control of a strong promoter, cytomegalovirus (CMV) promoter. Transfection of the expression vector into the RDFs is performed using Lipofectamine 3000 transfection reagent (Invitrogen, Thermo Fisher Scientific) according to the manufacturer’s instructions. The cells are incubated for 48 h post-transfection to allow for protein expression.

For the knockdown of VHL, SDF-1 and HIF-1α, specific shRNA targeting their mRNA was designed. The detail information of the shRNA was shown in Table 1. shRNA was cloned into a plasmid vector. For transient knockdown, transfect the cells with the shRNA using the Lipofectamine 3000 transfection reagent (Invitrogen). For stable knockdown, the cells were transduced with the lentiviral particles containing the shRNA construct.

**Table 1:** The detail information of shRNA

Gene names	No	Target seq (5'–3')
VHL	1	TCAGCAGAGAAAAATGAGAGA
	2	**TCCTTCCCGTCAGCCCCTAGC**
	3	TATACAGTTTTGTGGCCTGAT
HIF1α	1	**AGTTACAGGATTCCAGCAGAC**
	2	CTGATTGCATCTCCACCTTCT
	3	TGCTCAGAGGAAGCGAAAAAT
SDF-1	1	GCTACCAAAAGATCATCCTCA
	2	GTGCAATGGCCACTTAGCATC
	3	**GGGCGCCAAGTAACCTGCCAG**
ZNF667	1	**TTAACGTTTCCCTCTATAGA**
	2	GTCTGTAACTCCAGTTTCAGAG
	3	AGAGGTGCTGGTACATGCTGC

Note: the sequence with bold font was used in the formal experiment

### Quantitative polymerase chain reaction (qPCR)

From both cells and tissues, the total RNA was extracted using TRIzol reagent (KeyGEN BioTECH, KGA1201, China) and further reversed to cDNA using the First-strand cDNA synthesis kit from KeyGen BioTECH (KGA1316). qPCR was conducted using Realtime PCR Master Mix (SYBR Green) from KeyGen BioTECH (KGA1339). The pimers employed for qPCR were shown in the Table 2.

**Table 2:** The information of primers for PCR assay

Names	Direction	Sequence (5'—> 3')	Tm(°C)
NCOA1	Forward	AAAGGATCGCCATGTGACACG	63
	Reverse	TCTGCACGTCATCATCAGTCG	62.2
VHL	Forward	CTCAGCCCTACCCGATCTTAC	61.1
	Reverse	ACATTGAGGGATGGCACAAAC	60.9
SDF1	Forward	TGCATCAGTGACGGTAAACCA	61.7
	Reverse	TTCTTCAGCCGTGCAACAATC	61.4
ZNF667	Forward	GGGTGCCTCTTTCTCCTGAC	60
	Reverse	CAAGCCCCCAGCTTGTCTTA	59.9
Actb	Forward	CCCGCGAGTACAACCTTCTT	60.4
	Reverse	AACACAGCCTGGATGGCTAC	60.4
VHL promoter	Forward	TCAGAGGTGGCTTACGTGTC	59.4
	Reverse	AGCATACAGCTACCATCAGGTG	59.9
SDF1 promoter	Forward	TTTGCTGGTTTCTGCTTCGC	59.97
	Reverse	GGGCTCCACCTAGGGACTAA	60.3
Actb promoter	Forward	CCTGCCACAAGCCTAGAACA	59.96
	Reverse	CACACGGGGTCTGGATTGAA	59.96

### Western blot assay

Proteins were isolated from cells or tissues using RIPA buffer (Thermo Fisher) containing protease inhibitors. Equal amounts of protein samples were loaded onto a 10% SDS-PAGE gel for electrophoresis. The proteins were transferred from the gel to a nitrocellulose membrane (Bio-Rad) and then blocked in 5% non-fat dry milk to reduce non-specific binding. The membrane was incubated overnight at 4 °C with primary antibodies specific to the target proteins including ZNF667, NCOA1, VHL HIF-1α, SDF1, p-AKT, AKT, p-FAK, FAK, TGF-β, VEGFR-2, PECAM-1 and β-actin. The detail information of the antibodies is shown in Table 3. After washing with PBS-Tween, the membrane was incubated with a suitable HRP-conjugated secondary antibody for 1h. The blots were observed using a chemiluminescent substrate and X-ray film.

**Table 3:** The information of the primary antibodies

Protein name	Dilution	Company
ZNF667	1:50	Abcam
NCOA1	1:50	Abcam
VHL	1:50	Abcam
HIF-1α	1:50	Abcam
SDF1	1:100	Abcam
p-AKT	1:200	Abcam
AKT	1:200	Santa Cruz
p-FAK	1:100	Santa Cruz
FAK	1:100	Abcam
TGF-β	1:200	Abcam
VEGFR-2	1:200	Abcam
PECAM-1	1:100	Proteintech
β-actin	1:200	Proteintech

### Co-immunoprecipitation (Co-IP)

Cell lysates of RDFs were prepared using Co-IP kit (Beyotime Biotechnology, Shanghai, China), and subsequently mixed with primary antibodies against HIF-1α. A portion (10%) of the total cell lysate was saved as the input control. Protein A/G agarose beads (Beyotime Biotechnology) were added for affinity binding to the primary antibodies. The unbound antibodies were otherwise washed off through the sequential wash with PBS and cell lysis buffer. The agarose beads were resuspended in 20 µl 1 × SDS-PAGE loading buffer, and boiled for western blot analysis of the precipitated Ubiquitin and VHL protein.

### Dual luciferase reporter assay

Three predicted ZNF667-binding sites at the *VHL* gene promoter (Binding sites: 5’-GGATGAGCTCAG-3’; 5’-TTAAGAGCACTG-3’; 5’-TAAAAAGCTCA-3’) were individually cloned into pGL4 vector (Addgene, Inc., Cambridge, MA, USA) upstream of the firefly luciferase coding region within restriction sites XhoI and NotI (Takara Bio, Inc., Otsu, Japan). In addition, the predicted ZNF667-binding sites were mutant and also cloned into the pGL4 vector to construct VHL mutant type (MT) reporters. The mutant sequences were shown in Fig. 2G. The vectors were transfected to cells using Lipofectamine 2000 (Invitrogen). Cells were harvested at 48h and the activity of firefly luciferase was normalized to that of renilla luciferase.

### Chromatin immunoprecipitation (ChIP) assay

The assay was performed using a Magna ChIP Kit (Millipore, Bedford, MA, USA). In brief, nuclear DNA is fragmented into 500–1000 bp through sonication. The chromatin extract was incubated with antibody against MLL3, MLL4, SETD1A, EP300 (abcam) or IgG (Millipore). After immunoprecipitation, DNA–protein-antibody complex was separated and protein was removed. The purified DNA was then analyzed by using qRT-PCR. The primer information was shown in Table 2. A gene locus (Actinb promoter) was used as a unrelated control to demonstrate the specificity of the histone modifications and ZNF667 binding to the VHL promoter. The IgG group were used as the negative control.

### Electrophoretic mobility shift assay (EMSA)

EMSA was conducted to study the interaction between ZNF667 and *VHL* gene promoter. Biotin-labelled single-stranded DNA probe (WT probe: 5’-AGCAACCAGTGCTCTTAACCACTG-3’) was synthesized based on the *VHL* gene promoter that was specifically bound by ZNF667 protein. An unlabelled complementary DNA probe (cold probe) was synthesized to competitively bind to the labelled DNA probe with ZNF667 protein. As a control, a biotin-labelled mutant probe (5’-AGCAACCAGTACTGCGTACCACTG-3’) failed to bind to the WT probe. Nuclear protein was extracted from RDFs cells and mixed with the probes in an EMSA binding buffer (Thermo Fisher Scientific). The protein/DNA probe complex was subject to electrophoresis and transferring to nylon membrane. It was finally detected using HRP-conjugated streptavidin and visualized with ECL reagents. For supershift analysis, 200 ng of anti-ZNF667 antibody was added into the protein/DNA probe complex, followed by gel electrophoresis and ECL visualization.

### Cell viability assay

The viability of EPCs was evaluated using CCK8 reagent. Cells were cultured in a humidified incubator at 37 °C for indicated times. Absorbance at 450 nm was then surveyed by an automatic microplate reader (Thermo Scientific).

### Migration assays

The migratory capacity of EPCs was evaluated using a Transwell chamber assay. EPCs were seeded in the upper chamber and cultured with DMEM medium, and the lower chamber used EGM-2 medium as a chemoattractant. After incubation, the number of migrated cells is quantified.

In addition, cell scratch method was also used to evaluate the migration capacity. Cells were seeded in 6-well plates to grow to approximately 90% confluency. The cells were scraped with a pipette and then rinsed with serum-free 10% FBS medium for 24h. Cell migration was examined using phase contrast microscopy (Olympus, Tokyo, Japan).

### Tube formation assay

EPCs were initially cultured in the EGM-2 medium containing growth factors such as VEGF and EGF for 3 days to promote the differentiation to vascular endothelial cells. RDFs were cultured in DMEM medium under hypoxic conditions for 24h and under normoxic conditions for two hours. The culture medium collected and then added to EPCs for the tube formation. The ability of EPCs to form capillary-like structures is assessed using microscopy.

### HE staining

The distal third of the flap (approximately 1 × 3 cm) was defined as the primary region of interest (ROI) for histological analyses. Tissue slices were fixed in 4% paraformaldehyde at 4 °C for 30 min. After being rinsed with PBS, slices were stained with hematoxylin and eosin (H&E) using standard procedures.

### Quantification of endogenous EPC mobilization in blood and flap tissue

This study collected peripheral blood samples from rats in the Sham, I/R, and RIPC + I/R groups prior to any transfusion of labelled EPCs. Flow cytometry was used to quantified the population of circulating endogenous EPCs defined as cells positive for the surface markers CD34, and CD133. In addition, flow cytometry was used to quantify endogenous EPCs homing to the flap tissue prior to any transfusion of labelled EPCs. The entire flap was harvested, minced, and enzymatically digested into a single-cell suspension. Flow cytometry gated on live, nucleated cells and quantified the cells that were positive for CD34 and CD133. The results were shown as the percentage of CD34 + /CD133 + EPCs to the total living cells.

### In vivo imaging

DiR dye (working solution 2 μM) was used to label the isolated EPCs from peripheral blood of rat. After RIPC and I/R treatments, EPCs were injected to the rats through the tail vein (1 × 10 ^ 6/rat). *In vivo* imaging equipment (AV600; AniViev) was used to observe the amount of EPCs gathered in the skin flap area at 48 h after the flap surgery.

### Measurement of blood stream velocity

The blood stream velocity in the artery of flap was measured using Color Doppler Ultrasound with the Laser Doppler Flowmeter (MoorLDI-2, Moor Instruments Ltd, Axminster Devon, UK). After anesthesia in rats, the changes in blood flow and vascular morphology of the iliopsoas artery, thoracodorsal artery and intercostal artery were measured using a Doppler flowmeter, and the blood perfusion and flow velocity were recorded at 14 days after the flap surgery.

### Statistical analysis

Data analysis was performed using GraphPad Prism software. All quantitative data are expressed as mean ± SD. Normality was assessed using the Shapiro–Wilk test. For comparisons between two groups, an unpaired two-tailed Student's t-test was used. For comparisons among multiple groups under a single experimental factor, a one-way analysis of variance (ANOVA) was conducted, followed by Tukey’s post-hoc test for multiple comparisons. For experiments with a multi-factor design (e.g., treatment and time), a two-way ANOVA was performed, followed by Tukey's post-hoc test. The specific factors for each two-way ANOVA analysis are detailed in the corresponding figure legend. Statistical significance was defined as a *p*-value < 0.05. For primary endpoints, effect sizes (e.g., Cohen's d) and 95% confidence intervals for mean differences are reported. The sample size (n) for each experiment, defined as the number of biological or technical replicates, is provided in the figure legends.

## Results

### Genes changed by RIPC and I/R treatments in the flap transplantation surgery of rat

**Fig. 1:**
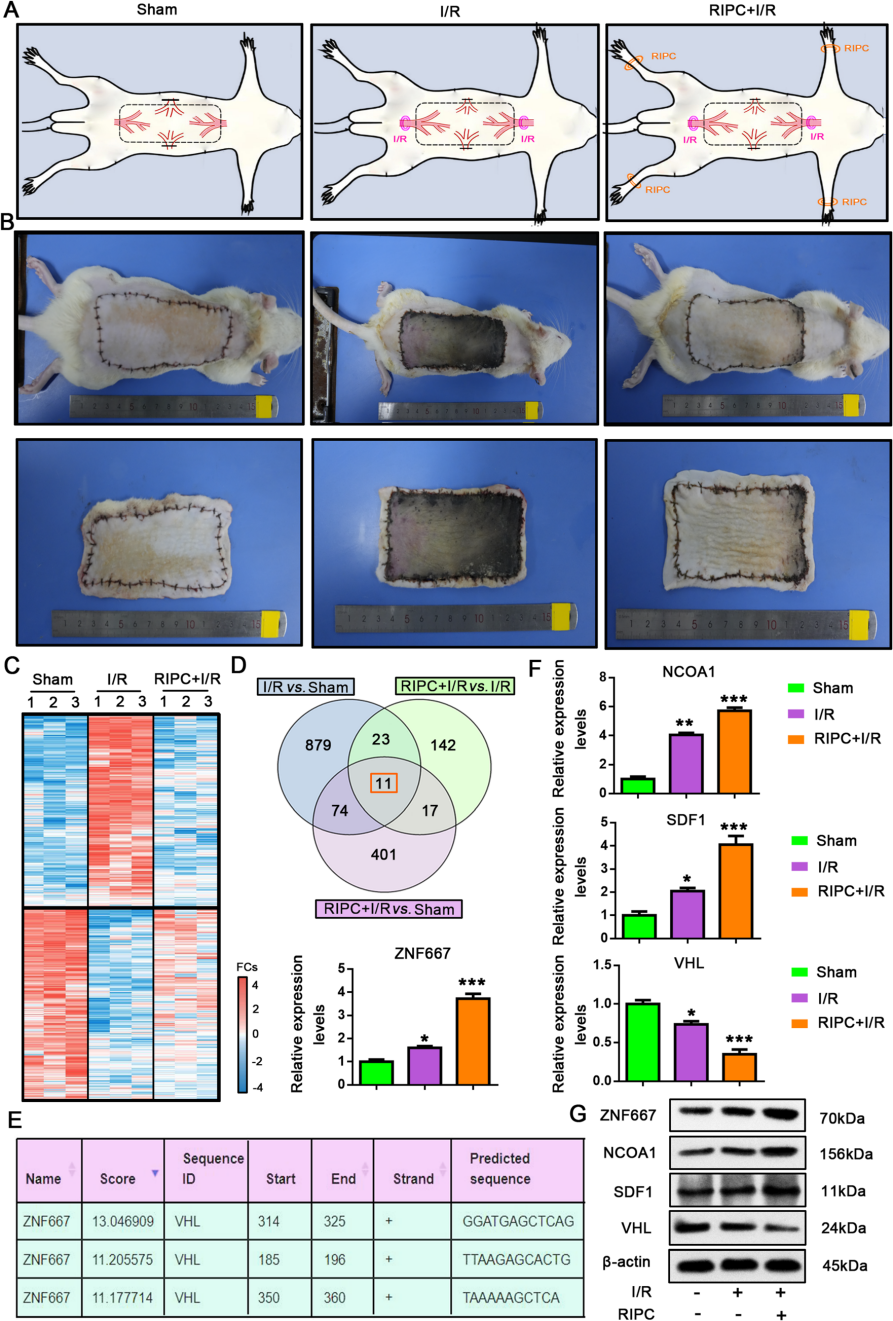
**Rat flap transplantation surgery and gene expression analysis.** **A**. Diagrammatic sketch of the rat flap surgery in the sham, I/R and RIPC + I/R group. **B**. Appearance of the flap on the seventh day in different groups. **C**. Schematic of high-throughput sequencing to find differentially expressed genes. **D**. Intersection analysis of differentially expressed genes among the three groups. **E**. Prediction of ZNF667 as a transcriptional inhibitor of VHL gene expression. **F**. PCR analysis of NCOA1, SDF1 and ZNF667 expression and VHL expression after I/R treatment. **G**. Western blot analysis of NCOA1, SDF1 and ZNF667 expression and VHL expression after I/R treatment. *p < 0.05 and ***p < 0.001 vs. sham (n = 6)

This study conducted flap transplantation surgery in rat. The intercostal artery was cut, but the iliopsoas artery and thoracodorsal artery were not severed in the sham group. The Schematic diagram was shown in Fig. 1A. The rat flap surgery was also conducted in I/R and RIPC + I/R groups. Differently, in these two groups, iliopsoas and thoracodorsal arteries were ligated for flap ischemia for 6h, and then the ligation was released for reperfusion. In RIPC + I/R group, each rat was subjected to the clamping at four limbs for 5 min and the release for 5 min. These processes were repeated three times as RIPC before I/R. Figure 1B shows the appearance of the flap on the seventh day. Compared to the sham group, I/R group had large area of necrosis in flap. However, the area of necrosis was much smaller in RIPC + I/R group than in I/R group. To understand the mechanism underlying the effects by I/R and RIPC treatment, this study conducted high-throughput sequencing to find genes that were differentially expressed among the three groups (Fig. 1C). A total of 11 genes were differentially expressed among the three groups, as indicated by the intersection analysis (Fig. 1D). Among the 11 genes, *NCOA1*, *SDF1* and *VHL* were known to participate in the angiogenesis. *ZNF667*, also in the 11 genes, was predicted to regulate *VHL* gene expression through serving as the transcriptional inhibitor (Fig. 1E). Jasper (https://jaspar.elixir.no/) showed the possible binding sites of ZNF667 at the promoter region of *VHL* gene. PCR and western blot analysis exhibited increased NCOA1, SDF1 and ZNF667 expression, but decreased VHL expression after I/R treatment (Fig. 1F and G). Treatment with RIPC before I/R further increased NCOA1, SDF1 and ZNF667 expression, but decreased VHL expression compared with I/R treatment alone.

### ZNF667 functions as a transcriptional inhibitor of vhl resulting in the increase of sdf1 in fibroblasts

**Fig. 2:**
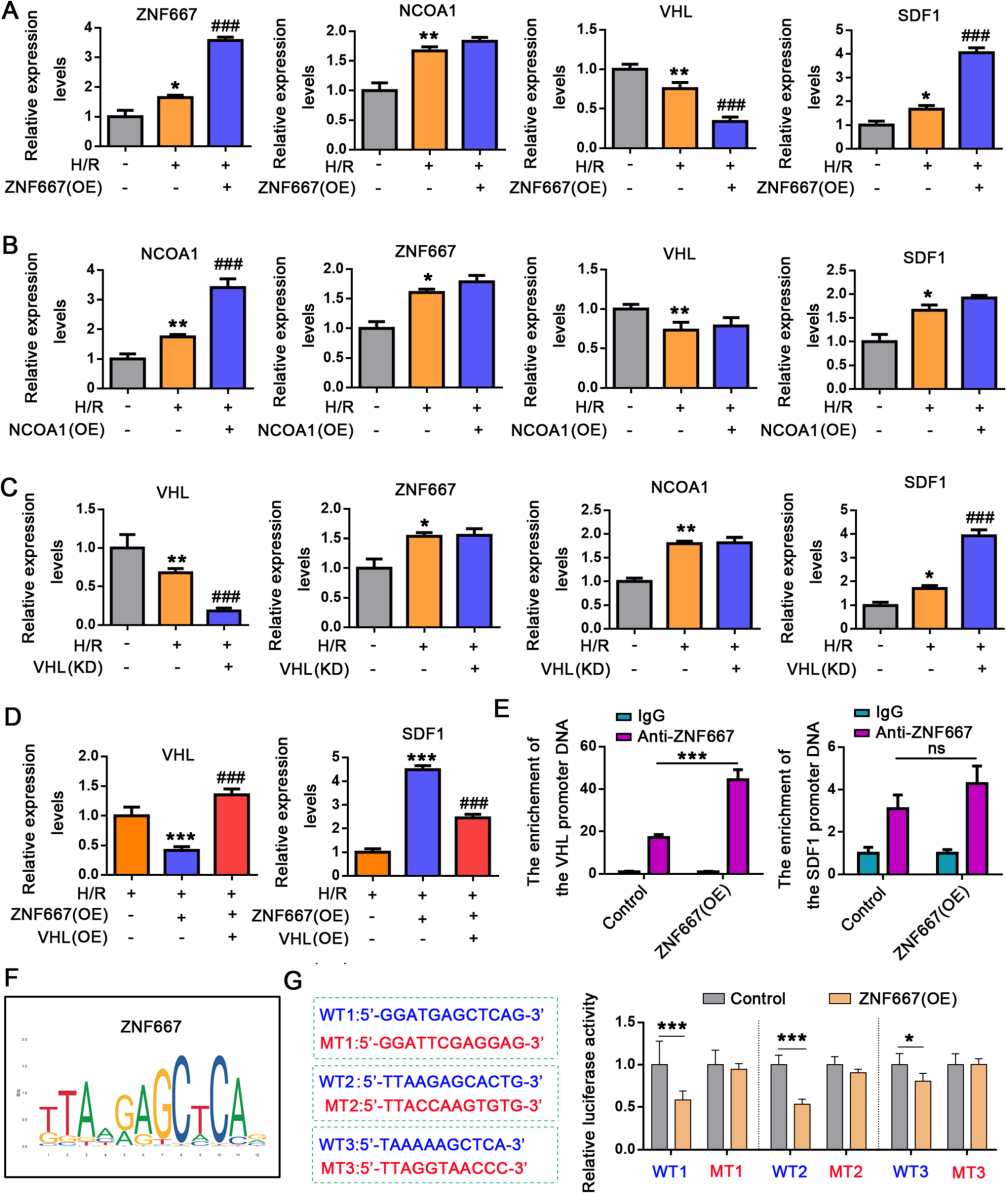
**ZNF667 functions as a transcriptional inhibitor of VHL resulting in the increase of SDF1 in fibroblasts.** **A**. PCR analysis of ZNF667 expression in RDFs exposed to H/R. **B**. PCR analysis of the effects of ZNF667 overexpression and NCOA1 overexpression on ZNF667, VHL, and SDF1 expression in RDFs under H/R conditions. **C**. PCR analysis of the effect of VHL knockdown on ZNF667, NCOA1, and SDF1 expression in RDFs under H/R conditions. **D**. Reversal of SDF1 increase by ZNF667 overexpression with VHL overexpression. **E**. ChIP assay showing ZNF667 binding to VHL gene promoter. **F**. The specific DNA sequence recognized by ZNF667. **G**. Luciferase report assay showing the effect of ZNF667 overexpression on the luciferase report activity of WT and MT constructors at the VHL gene promoter. * *p* < 0.05, **p < 0.01 and ***p < 0.001 vs. control (n = 3); ###*p* < 0.001 vs. H/R or ZNF667(OE) (n = 3)

Study *in vitro* overexpressed ZNF667 and NCOA1 and knocked down VHL before RDFs subjected to H/R treatments to mimic the effects of I/R and RIPC treatments *in vivo*. Exposing to H/R increased ZNF667 expression in RDFs as indicated by PCR (Fig. 2A). Transfection of the overexpression vector further increased ZNF667 expression in RDFs upon H/R. Although, NCOA1 was also increased in RDFs upon H/R, ZNF667 overexpression failed to further increased NCOA1. It suggests that NCOA1 is no regulated by ZNF667. H/R treatments resulted in the reduction of VHL expression and the increase of SDF1 expression in RDFs. ZNF667 overexpression further enhanced the effects by H/R treatments. PCR analysis showed that overexpression of NCOA1 had no effect of ZNF667, VHL and SDF1 expression in RDFs under H/R conditions (Fig. 2B). VHL knockdown had no effect of ZNF667 and NCOA1, while increased SDF1 expression in RDFs under H/R conditions (Fig. 2C). These results suggested that ZNF667 suppresses VHL expression; the reduction of VHL expression conversely increased SDF1 expression. Indeed, the increase of SDF1 by ZNF667 overexpression was reversed by VHL overexpression (Fig. 2D). ChIP assay revealed that ZNF667 bound to *VHL* gene promoter, but ZNF667 was unable to bind to *SDF1* gene promoter (Fig. 2E). ZNF667 specifically recognizes the DNA sequence ‘TTAAGAGCTCA’ (Fig. 2F). Based on the prediction of the Jasper, we constructed three WT luciferase constructors and corresponding MT constructors. Luciferase report assay showed that ZNF667 overexpression decreased the luciferase report activity of the three WT constructors with the most significant effect on the WT1 and WT2 (Fig. 2G). The luciferase report activity of the three MT constructors was not impacted by ZNF667 overexpression. The results suggested that ZNF667 binds to the three locations at the *VHL* gene promoter, whereby suppressing the transcription.

### The suppression VHL by ZNF667 promotes the hif-1α-mediated SDF1 expression in fibroblasts upon H/R treatments

**Fig. 3:**
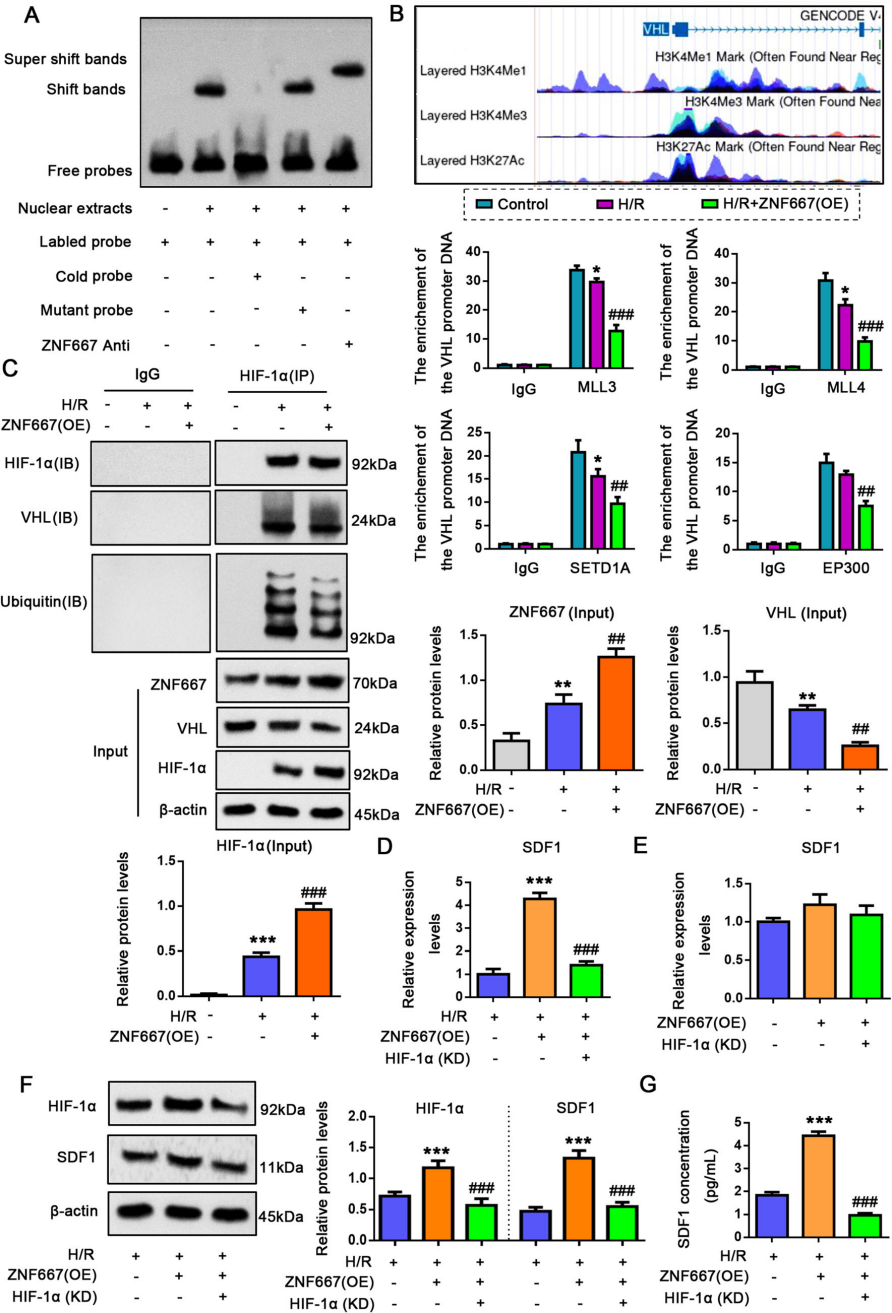
**The suppression VHL by ZNF667 promotes the Hif-1α-mediated SDF1 expression in fibroblasts upon H/R treatments.** **A**. EMSA assay confirming the binding of ZNF667 to VHL gene promoter. **B**. ChIP assay showing the suppression of the binding of MLL3/4, SETD1A, and EP300 to VHL gene promoter by ZNF667 overexpression. *p < 0.05 vs. control (n = 3); ##*p* < 0.01 and ###*p* < 0.001 vs. H/R (n = 3). **C**. Western blot assay showing the band of HIF-1α in RDFs after H/R treatment. **p < 0.01, ***p < 0.001 vs. control(n = 3); ##*p* < 0.01 and ###*p* < 0.001 vs. H/R (n = 3). **D**. PCR analysis showing the abolition of the increase of SDF1 by ZNF667 overexpression after H/R treatment with HIF-1α Knockdown. ***p < 0.001 vs. H/R (n = 3); ###*p* < 0.001 vs. ZNF667(OE) (n = 3). **E**. PCR analysis showing that SDF1 expression is not changed by ZNF667 overexpression alone or in combination with HIF-1α Knockdown under normoxic conditions. **F**. Western blot analysis showing the effect of HIF-1α Knockdown on the increase of HIF-1α and SDF1 protein levels by ZNF667 overexpression under H/R conditions. ***p < 0.001 vs. H/R (n = 3); ###*p* < 0.001 vs. ZNF667(OE) (n = 3). **G**. ELISA assay showing the increase of SDF1 in the culture medium of RDFs exposed to H/R treatments by ZNF667 overexpression and the prevention of this increase with HIF-1α Knockdown. ***p < 0.001 vs. H/R (n = 3); ###*p* < 0.001 vs. ZNF667(OE) (n = 3)

This study conducted the EMSA assay to further verify the binding of ZNF667 to *VHL* gene promoter. The shift band was shown when labeled *VHL* probe was mixed with the nuclear extract. The shift band was disappeard after adding enough cold probe to the labeled *VHL* probe and nuclear extract. Adding the mutant probe had no such effect. Adding anti-ZNF667 antibody to the mixture of labeled *VHL* probe and nuclear extract led to the appearance of the super shift band. These results collectively confirm that ZNF667 binds to *VHL* gene promoter (Fig. 3A). Bioinformatics analysis by UCSC Genome (http://www.genome.ucsc.edu/cgi-bin/hgGateway? redirect = manual&source = www.genome.ucsc.edu) implies that VHL transcription is regulation by histone methylation at H3K4Me1 and H3K4Me3 and by histone acetylation at H3K27Ac. These histone methylation and acetylation generally promote gene transcription and expression. MLL3/4, SETD1A and EP300 have been identified for the histone methylation and acetylation. Through the ChIP assay, we noticed that ZNF667 overexpression suppressed the binding of MLL3/4, SETD1A and EP300 to *VHL* gene promoter (Fig. 3B). Therefore, the transcriptional inhibition by ZNF667 is probably associated to hinder the binding of MLL3/4, SETD1A and EP300 to *VHL* gene promoter for the histone methylation and acetylation. It is well-known that VHL functions as the important E3 ubiquitin-protein ligase for the ubiquitination-mediated degradation of HIF-1α. HIF-1α is almost undetectable under normoxic conditions due to rapid degradation by VHL-induced ubiquitination. CO-IP with western blot assays exhibited the band of HIF-1α in RDFs after H/R treatment (Fig. 3C). ZNF667 overexpression decreased the total VHL protein level and the amount of VHL binding to HIF-1α. It was correlated with the decreased ubiquitination of HIF-1α and increased protein levels of HIF-1α. Therefore, ZNF667 positively regulates HIF-1α protein level, through suppressing VHL-mediated ubiquitination. It has been identified that SDF1 is a target of transcript factor. To determine that the promoting effect of SDF1 by ZNF667 is dependent on the HIF-1α, this study knocked down HIF-1α. PCR analysis showed that the increase of SDF1 by ZNF667 overexpression after H/R treatment was abolished with HIF-1α knockdown (Fig. 3D). Under normoxic conditions, SDF1 expression was not changed by ZNF667 overexpression alone or in combination with HIF-1α knockdown (Fig. 3E). The results also supported the conclusion that the promoting effect of SDF1 by ZNF667 is dependent on the HIF-1α. As indicated by western blot, HIF-1α and SDF1 protein levels were increased under H/R conditions by ZNF667 overexpression. HIF-1α knockdown prevented the increase of SDF1 by ZNF667 overexpression (Fig. 3F). Elisa assay was conducted to detect RDFs-secreted SDF1 in the culture medium. ZNF667 overexpression increased SDF1 in the culture medium of RDFs exposing to H/R treatments. HIF-1α knockdown also prevented the increase of SDF1 in the culture medium (Fig. 3G).

### ZNF667 overexpression in fibroblasts promotes EPCs proliferation and differentiation

**Fig. 4:**
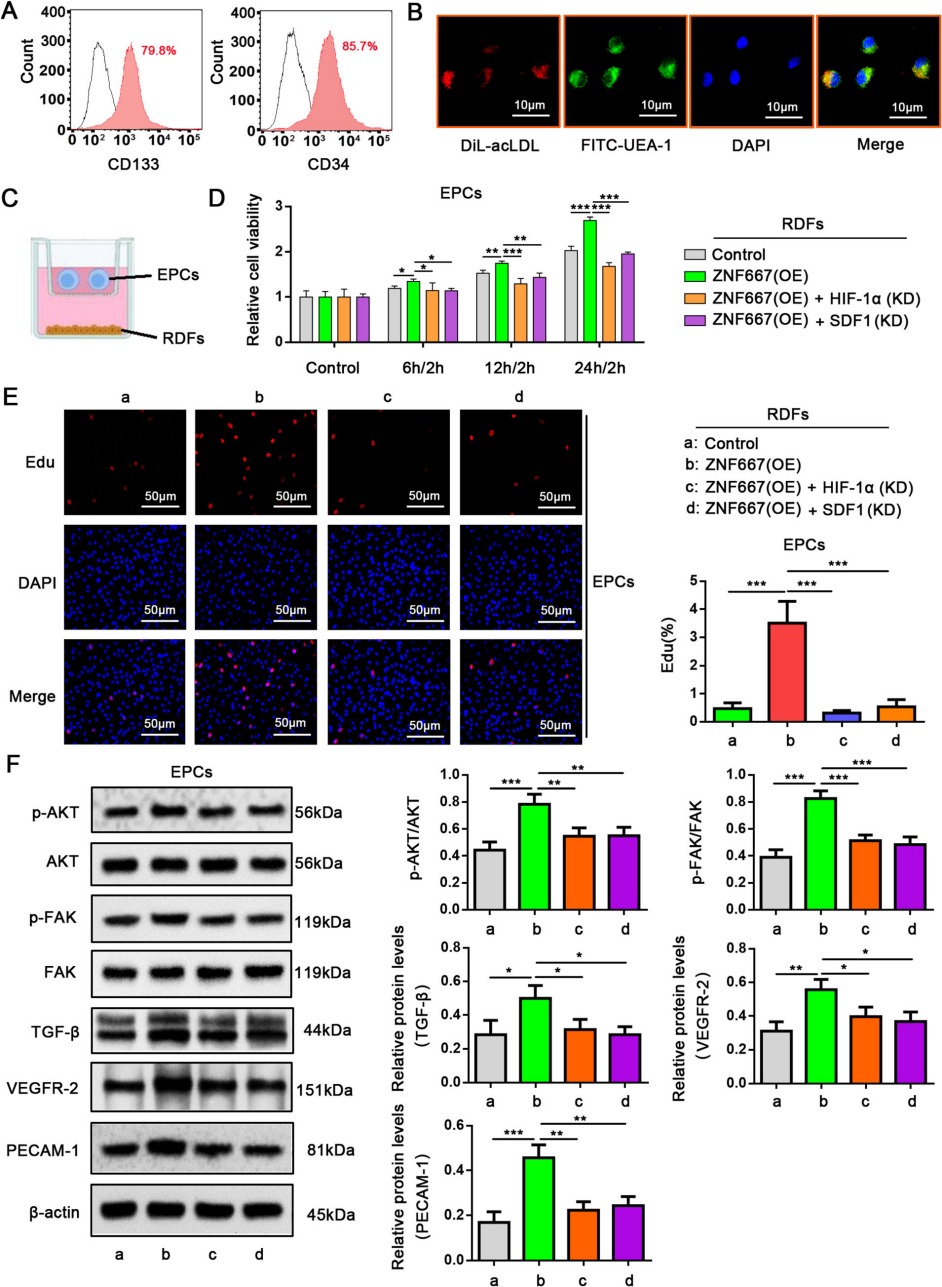
**ZNF667 overexpression in fibroblasts promotes EPCs proliferation and differentiation.** **A**. Flow Cytometry verification of isolated EPCs by detecting the biomarkers of CD133 and CD34. **B**. Characterization of isolated EPCs by the uptake of DiI-acLDL and FITC-UEA-1. **C**. Diagrammatic sketch of the co-culture system of EPCs and RDFs. **D**. Increase in the cell viability of EPCs when cultured with ZNF667-overexpressing RDFs compared to non-treated RDFs. **E**. Increase in the proliferation rate of EPCs when cultured with ZNF667-overexpressing RDFs compared to non-treated RDFs. **F**. Western blot assay showing the elevation of the phosphorylation levels of AKT and FAK and the protein levels of TGF-β in EPCs by ZNF667 overexpression in RDFs. * *p* < 0.05, **p < 0.01 and ***p < 0.001 (n = 3)

This study isolated EPCs from the peripheral blood of rats. The isolated EPCs were verified by Flow Cytometry detecting the biomarkers of CD133 and CD34 (Fig. 4A). In addition, the isolated EPCs were characterized by the uptake of DiI-acLDL and FITC-UEA-1 (Fig. 4B). EPCs were co-cultured with RDFs in DMEM culture medium using transwell system. Figure 4C shows the diagrammatic sketch of the co-culture system. Both cells are maintained in a hypoxic chamber with low oxygen concentration (1% O_2_) for 6, 12 or 24h. After the hypoxic treatment, cells are returned to normoxic conditions for further culture for two hours. The cell viability of EPCs was increased when they were cultured with ZNF667-overexpressing RDFs compared to be cultured with non-treated RDFs (Fig. 4D). Knockdown of HIF-1α and SDF1 in ZNF667-overexpressing RDFs prevented the increase of EPCs’ viability. Edu staining was conducted to detect the proliferation rate of EPCs. Similarly, the proliferation rate of EPCs was increased when they were cultured with ZNF667-overexpressing RDFs compared to be cultured with non-treated RDFs (Fig. 4E). Knockdown of HIF-1α and SDF1 in ZNF667-overexpressing RDFs prevented the increase of EPCs’ proliferation. The results suggest that ZNF667-overexpressing RDFs promote the viability and proliferation of EPCs through SDF1.

It is known that SDF1 functions as a chemoattractant (ligand) binding to the G-protein coupled receptor, chemokine receptor 4 (CXCR4) which is expressed on the EPCs. SDF1/CXCR4 signal plays important roles of the proliferation, migration and differentiation of EPCs through stimulating signaling molecules such as AKT, FAK and TGF-β. Western blot assay exhibited that ZNF667 overexpression in RDFs elevated the phosphorylation levels of AKT and FAK and the protein levels of TGF-β in EPCs (Fig. 4F). Knockdown of HIF-1α and SDF1 in ZNF667-overexpressing RDFs prevented the increase of p-AKT, p-FAK and TGF-β in EPCs. VEGFR-2 and PECAM-1 are biomarkers of vascular endothelial cells. Therefore, their expression levels can be used evaluate the degree of EPCs differentiation to vascular endothelial cells. ZNF667 overexpression in RDFs promoted VEGFR-2 and PECAM-1 expression in EPCs, suggesting a higher degree of EPCs differentiation to vascular endothelial cells. The effect by ZNF667 overexpression was abolished with the knockdown of HIF-1α and SDF1.

### ZNF667 overexpression in fibroblasts promotes the migration and tube formation of EPCs

**Fig. 5:**
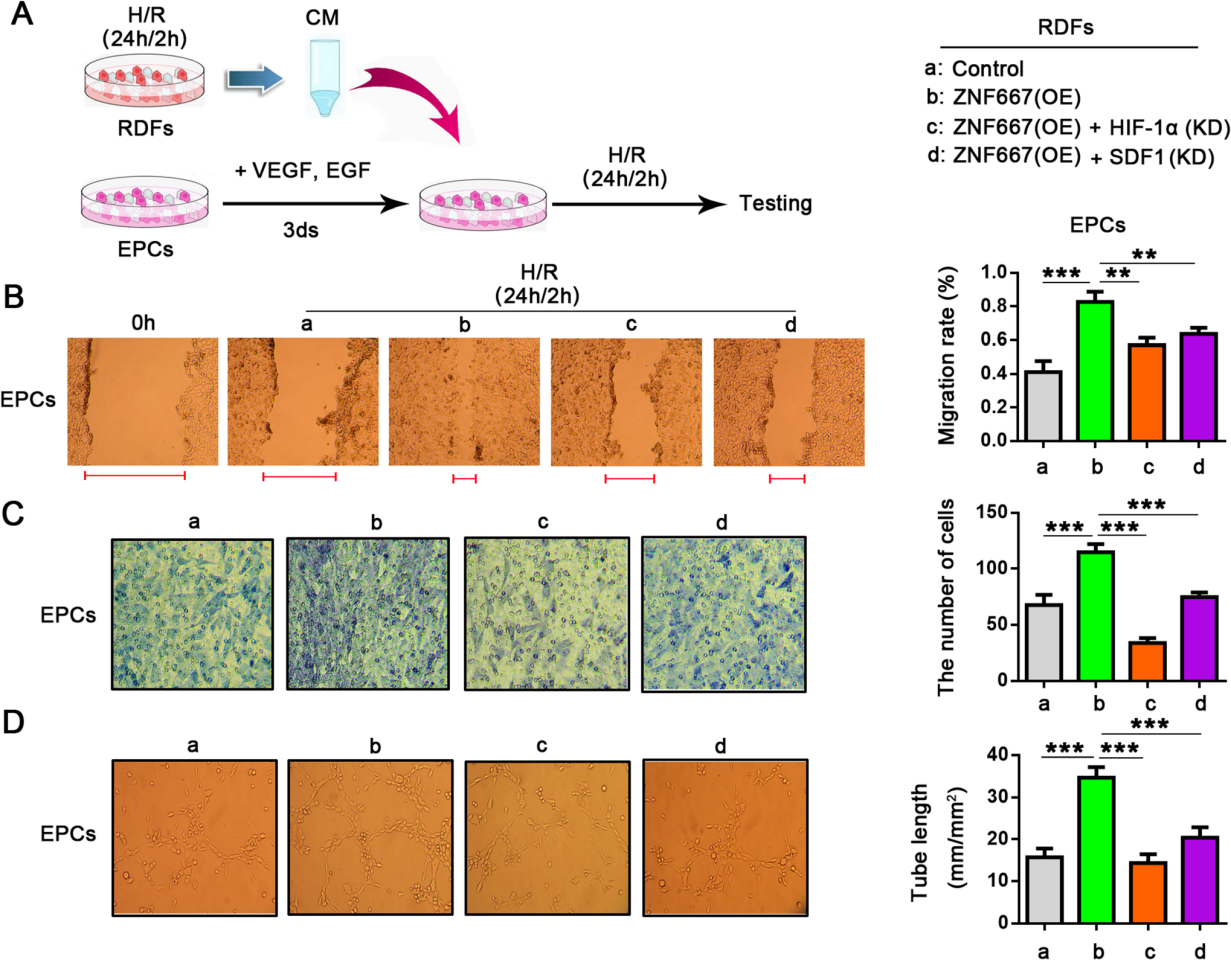
**ZNF667 overexpression in fibroblasts promotes the migration and tube formation of EPCs.** **A**. Diagrammatic sketch of the treatments for EPCs migration and tube formation assays. **B**. Evaluation of the migration capacity of EPCs by the cell scratch method. **C**. Evaluation of the migration capacity of EPCs by the transwell method. **D**. Promotion of the tube formation of EPCs by the culture medium of ZNF667-overexpressing RDFs and the prevention of this effect after HIF-1α and SDF1 Knockdown. **p < 0.01 and ***p < 0.001 vs. control (n = 3)

EPCs were initially cultured in the EGM-2 medium containing growth factors such as VEGF and EGF for 3 days to promote the differentiation to vascular endothelial cells. RDFs were cultured in DMEM medium under hypoxic conditions for 24h and under normoxic conditions for two hours. The culture medium collected and then added to EPCs for the migration and tube formation assays. The diagrammatic sketch of the treatments is shown in Fig. 5A. The migration capacity of EPCs were evaluated by two methods, namely cell scratch and transwell methods. Both methods showed that the migration capacity of EPCs was enhanced by the culture medium of ZNF667-overexpressing RDFs compared to that of non-treated RDFs (Fig. 5B, C). Knockdown of HIF-1α and SDF1 in ZNF667-overexpressing RDFs prevented the increase of EPCs’ migration capacity. The culture medium of ZNF667-overexpressing RDFs promoted the tube formation of EPCs, which was not observed after HIF-1α and SDF1 knockdown in ZNF667-overexpressing RDFs (Fig. 5D).

### RIPC treatment induces the accumulation of EPCs and angiogenesis, thereby preventing necrosis in the flap of rats

This study assessed the endogenous EPC in blood. We collected peripheral blood samples from rats in the Sham, I/R, and RIPC + I/R groups prior to any transfusion of labelled EPCs. As indicated by flow cytometry, I/R group had increased number of circulating endogenous CD34^+^/CD133^+^ EPCs compared to the sham group; RIPC treatment further increased the number of circulating CD34^+^/CD133^+^ EPCs compared to the I/R group, confirming that RIPC actively mobilizes endogenous EPCs from the bone marrow into the bloodstream (Supplementary Fig. 1A). In addition, we assessed the endogenous EPC homing in flap tissue by flow cytometry. As shown in Supplementary Fig. 1B, I/R group had increased number of CD34^+^/CD133^+^ EPCs compared to the sham group; RIPC pretreatment led to further increase in the number of endogenous EPCs homing to the flap compared to the I/R group alone.

**Fig. 6:**
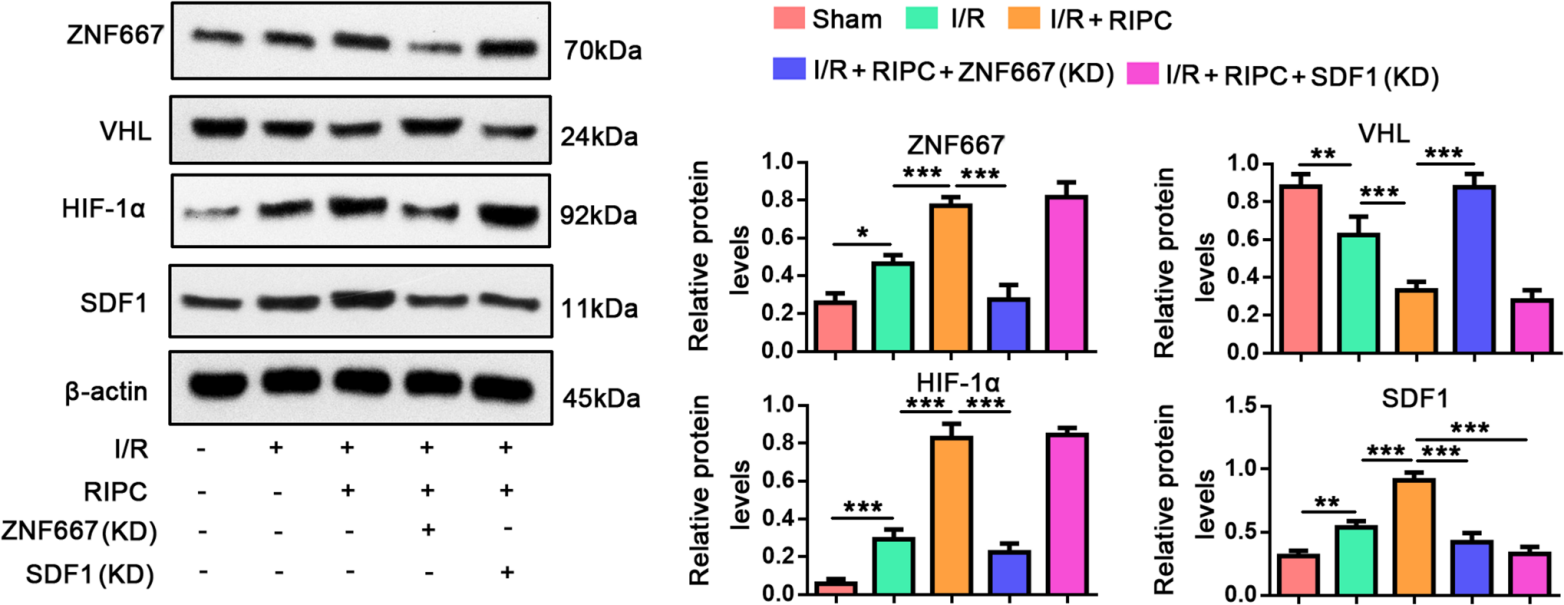
**Identification of The ZNF667-VHL-HIF-1α-SDF1 axis in rat flap model.** Western blot assay of ZNF667, VHL, HIF-1α, and SDF1 protein levels in the flap of the three groups 14 days after the surgery. *p < 0.05, **p < 0.01 and ***p < 0.001 (n = 6)

This study tested the protein levels of ZNF667, VHL, HIF-1α and SDF1 in the flap of the three groups through western blot assay 14 days after the surgery. Compared to the sham group, the I/R group showed increased ZNF667, HIF-1α and SDF1 and decreased VHL protein level (Fig. 6). RIPC + I/R group had higher ZNF667, HIF-1α and SDF1 protein levels and lower VHL protein level than I/R group. In RIPC + I/R group, the lentiviral particles containing the shRNA construct were injected to the flap to directly down-regulate ZNF667 and SDF1 protein levels. The down-regulation of ZNF667 caused the increase of VHL and the reduction of HIF-1α and SDF1. HE staining exhibited that RIPC + I/R treatments had higher microvessel density in the flap than I/R treatments (Fig. 6).

**Fig. 7:**
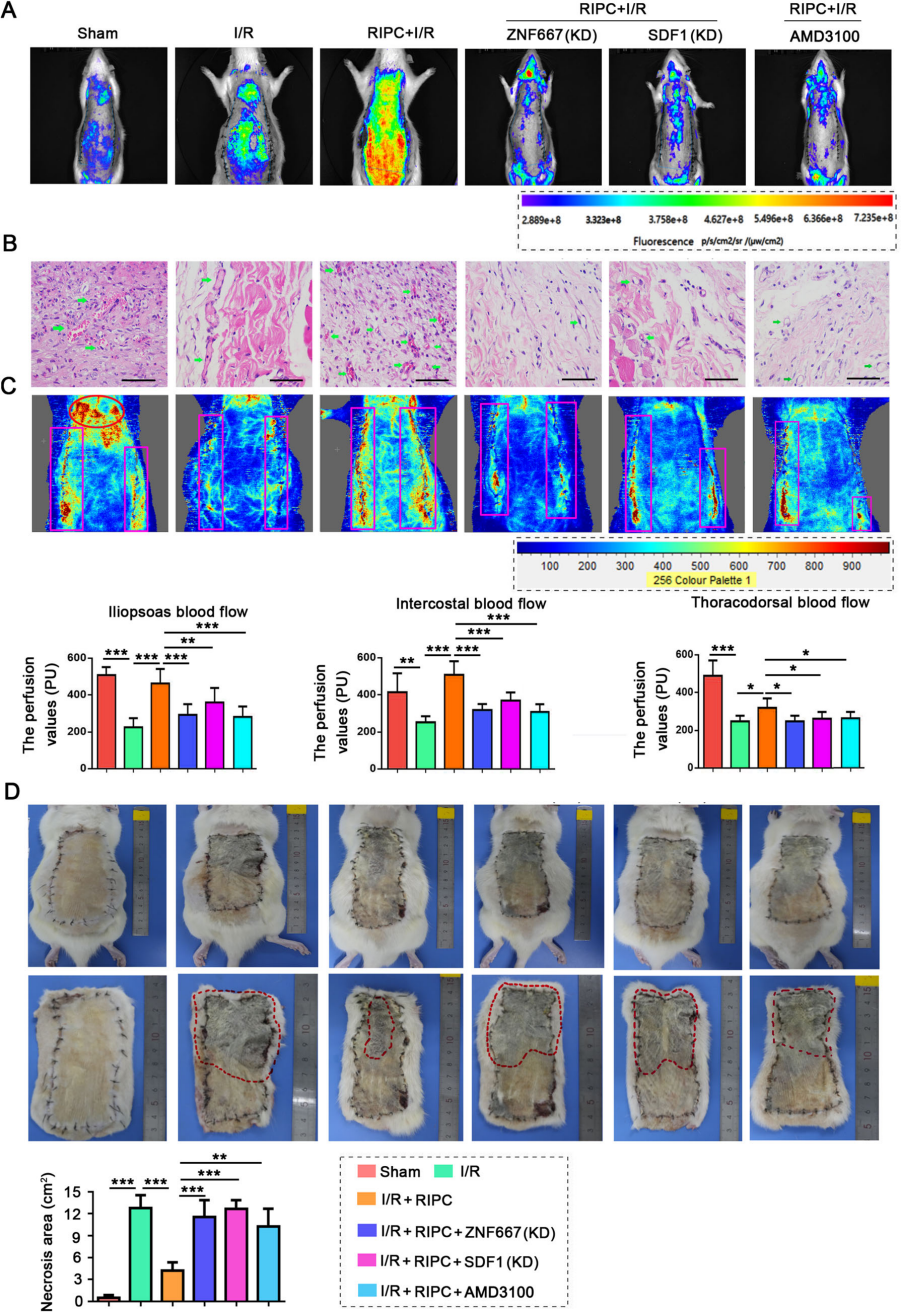
**RIPC treatment induces the accumulation of EPCs and angiogenesis, thereby preventing the necrosis in the flap of rats.** **A**. *In vivo* imaging detection of the amount of EPCs gathered in the skin flap area in different groups. **B**. HE staining showing the microvessel density in the flap of different groups. **C**. Measurement of blood flow velocity of the iliopsoas artery, thoracodorsal artery, and intercostal artery using a Doppler flowmeter. **D**. Comparison of the necrosis area in the flap of rats in different groups. * *p* < 0.05, **p < 0.01 and ***p < 0.001 vs. control (n = 6)

This study validated the role of the ZNF667-SDF1-CXCR4 axis in mediating the effects of RIPC on EPC homing to skin flaps and subsequent angiogenesis. *In vivo* imaging was used to quantify DiR-labeled EPCs in the flap region, with free DiR dye as a negative control. As expected, no significant fluorescence signal was detected at the flap site after free DiR injection (Supplementary Fig. 1C), because the unbound dye rapidly binds to serum proteins and becomes widely distributed, resulting in a diffuse background signal below the detection threshold in the specific area of interest. Compared with the sham group, I/R significantly increased the recruitment of DiR-labeled EPCs to the skin flap (Fig. 7A), an effect that was further enhanced by RIPC pretreatment. However, knockdown of ZNF667 or SDF1, as well as treatment with the CXCR4 antagonist AMD3100, suppressed EPC accumulation in the RIPC + I/R group. Consistent with these findings, HE staining revealed that microvessel density in the flap was higher in the RIPC + I/R group than in the I/R group (Fig. 7B). This increase was reversed by knockdown of ZNF667 or SDF1, or by treatment with AMD3100. Doppler flowmetry showed that blood flow velocity in the iliopsoas, thoracodorsal, and intercostal arteries was lower in the I/R group compared with the sham group (Fig. 7C). RIPC significantly improved arterial flow velocity after I/R injury, but this benefit was abolished by knockdown of ZNF667 or SDF1 or by AMD3100 treatment. Accordingly, RIPC before I/R reduced flap necrosis compared with I/R alone, and this protective effect was attenuated by ZNF667/SDF1 knockdown or AMD3100 administration (Fig. 7D).

## Discussion

This study was designed to investigate the role of RIPC in flap transplantation and elucidate its underlying molecular mechanisms. Our findings demonstrated that RIPC significantly enhanced angiogenesis and attenuated necrotic area formation in the flap, suggesting a protective effect against IRI. Furthermore, transcriptomic analysis revealed a set of differentially expressed genes (DEGs) following RIPC treatment, including *NCOA1*, *SDF1*, *VHL*, and *ZNF667*. Among these, several genes are functionally implicated in angiogenesis and tissue repair. For instance, NCOA1 has been reported to facilitate angiogenesis in breast tumors by co-activating HIF1α- and AP-1-mediated transcriptional upregulation of VEGFa [17]. Similarly, SDF1 (CXCL12) is a well-characterized chemokine that plays a pivotal role in angiogenesis and cell migration, both of which are essential for wound healing and tissue regeneration [18, 19]. Additionally, VHL, an E3 ubiquitin ligase responsible for the proteasomal degradation of HIF-1α, was found to be downregulated following RIPC treatment. This downregulation may lead to stabilization of HIF-1α, a key transcriptional regulator that drives the expression of pro-angiogenic factors, including VEGF [20, 21]. Consequently, HIF-1α-mediated signaling appears to be a critical mechanistic pathway through which RIPC exerts its angiogenic and tissue-reparative effects.

The biological function of ZNF667 in angiogenesis during tissue repair in skin flap remains poorly understood. Previous studies have reported that ZNF667 promotes angiogenesis following myocardial ischemia by mechanistically suppressing the expression of the anti-angiogenic gene *VASH1* through direct binding to its promoter region [22]. In the context of skin flap transplantation, ZNF667 has been demonstrated to mitigate leukocyte-endothelial adhesion after RIPC via the downregulation of P-selectin [10]. Furthermore, ZNF667 exhibits anti-inflammatory properties in macrophages, where its overexpression suppresses LPS-induced production of pro-inflammatory cytokines, including IL-1β, IL-6, and TNF-α [23]. Our study revealed a significant upregulation of ZNF667 in flap tissues following RIPC treatment. Importantly, genetic knockdown of ZNF667 impaired RIPC-induced angiogenesis and tissue repair, underscoring its critical role in these processes. Collectively, these findings highlight the pivotal contribution of ZNF667 to RIPC-mediated angiogenesis and tissue regeneration.

This study demonstrated that ZNF667 mediates the downregulation of VHL in skin flap tissues following RIPC treatment. While ZNF667 is known to function as a transcriptional repressor by binding to gene promoters, the precise mechanism underlying transcriptional suppression remained unclear. Our in-depth mechanistic investigation revealed that ZNF667 binding to the VHL promoter competitively inhibits the recruitment of histone-modifying enzymes, including MLL3/4, SETD1A, and EP300, to the same locus. Given that MLL3/4 and SETD1A are histone methyltransferases (catalyzing H3K4me1 and H3K4me3) and EP300 is a histone acetyltransferase (mediating H3K27ac), their exclusion from the VHL promoter leads to reduced histone methylation and acetylation—epigenetic modifications that are critical for transcriptional activation [24, 25]. Furthermore, *in vitro* experiments confirmed that ZNF667 overexpression suppresses VHL expression, which subsequently stabilizes HIF-1α protein levels under H/R conditions. These findings collectively suggest that ZNF667-mediated epigenetic silencing of VHL represents a key regulatory mechanism contributing to RIPC-induced tissue protection.

It is well established that HIF-1α stabilization occurs under hypoxic conditions due to impaired hydroxylation, which prevents VHL-mediated recognition and subsequent proteasomal degradation. Under normoxic conditions, prolyl hydroxylation of HIF-1α facilitates its binding to VHL, an E3 ubiquitin ligase that targets HIF-1α for ubiquitination and degradation. In the context of skin flap transplantation, the initial ischemic phase induces HIF-1α accumulation, thereby activating downstream transcriptional programs. However, subsequent reperfusion results in HIF-1α destabilization, ultimately attenuating its pro-angiogenic effects. Our findings demonstrate that RIPC maintains HIF-1α stability during the I/R cycle through ZNF667-mediated epigenetic suppression of VHL expression. This regulatory mechanism sustains HIF-1α protein levels in flap tissues following I/R injury, thereby preserving its angiogenic potential.

This study demonstrates that RIPC treatment promotes EPCs recruitment in skin flap tissues through the ZNF667/HIF-1α/SDF1 signaling axis. The mobilization and homing of EPCs play a pivotal role in RIPC-induced neovascularization and angiogenic processes during flap repair. Genetic ablation of either ZNF667 or SDF1 not only abolished RIPC-mediated EPC accumulation but also compromised neovascularization, ultimately resulting in tissue necrosis. These findings are consistent with established literature underscoring the critical contribution of EPCs to angiogenesis and tissue regeneration [26, 27].

Our findings primarily delineate a local signaling axis within the flap tissue, wherein RIPC-triggered upregulation of ZNF667 in dermal fibroblasts epigenetically suppresses VHL, leading to HIF-1α stabilization and subsequent SDF1 secretion. This cascade creates a pro-angiogenic microenvironment that promotes EPC functions. However, the initiation of this local response is likely dependent on systemic factors released by the remote ischemic stimulus. The well-established paradigm for RIPC involves the liberation of humoral factors (e.g., adenosine, bradykinin, or microRNAs) or neural signals from the preconditioned limb into the circulation [28], which then transmit protective messages to distant organs. This systemic mechanism is corroborated by our data showing the crucial role of EPC recruitment, as the systemically administered CXCR4 antagonist AMD3100, which blocks EPC mobilization and homing, abolished the therapeutic benefits of RIPC. Therefore, we propose a coordinated model where RIPC's effects are bimodal: a systemic phase involving the release of mediators from the preconditioned limb and the mobilization of bone marrow-derived EPCs, and a local phase within the target flap wherein these systemic signals activate the ZNF667-VHL-SDF1 axis, which in turn acts as a beacon for the recruited EPCs to facilitate angiogenesis. This synergy between systemic signaling and local amplification likely underlies the efficacy of RIPC in enhancing flap survival.

A potential limitation of our study is the use of shRNA-mediated knockdown for the *in vivo* functional validation of ZNF667 and SDF1. Although shRNA is a powerful tool, the possibility of off-target effects can never be entirely ruled out. However, several lines of evidence strongly suggest that the observed phenotypes—specifically, the impairment of angiogenesis and the abolition of RIPC's protective effect—are primarily attributable to the on-target knockdown of the intended genes. First, to minimize off-target potential, we selected shRNA sequences with high predicted specificity and validated their efficacy *in vitro* prior to *in vivo* application. Second, and more importantly, the knockdown of ZNF667 produced a highly coherent and logical cascade of molecular events: it led to the upregulation of its direct transcriptional target, VHL, which subsequently resulted in the decreased stability of HIF-1α and the downregulation of SDF1 expression. The precise molecular rescues the entire hypothesized ZNF667-VHL-HIF-1α-SDF1 axis. The convergence of our loss-of-function data with the gain-of-function and mechanistic studies in fibroblasts provides strong, multi-faceted support for our conclusions. Nevertheless, future studies employing more specific genetic tools, such as CRISPR/Cas9-mediated gene knockout, could provide further unequivocal confirmation of these findings.

**Fig. 8:**
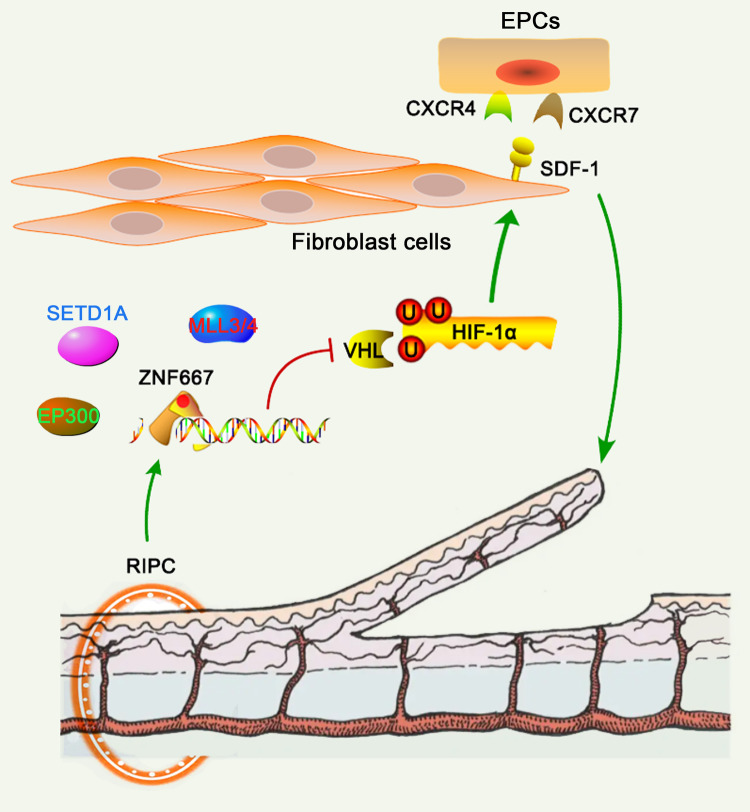
**Mechanism diagram of RIPC enhances flap survival via ZNF667/SDF1-mediated endothelial progenitor cells functions for angiogenesis**. Remote Ischemic Preconditioning (RIPC) triggers the increase of ZNF667 in the Flap. ZNF667 functions as a transcriptional inhibitor of VHL. In detail, ZNF667 binds to the VHL gene promoter, suppressing the binding of MLL3/4, SETD1A, and EP300, thereby inhibiting histone methylation and acetylation and reducing VHL transcription. ZNF667 decreases VHL-mediated ubiquitination, positively regulating HIF-1α protein level. The promoting effect of ZNF667 on SDF1 is dependent on HIF-1α. ZNF667-overexpressing RDFs promote the viability, proliferation, migration, and tube formation of Endothelial Progenitor Cells (EPCs) through SDF1. In summary, RIPC regulates the ZNF667-VHL-SDF1 axis and the functions of EPCs, enhancing angiogenesis and improving flap survival

Overall, our findings demonstrate that RIPC enhances flap survival by activating the ZNF667-VHL-HIF-1α-SDF1 axis, which in turn augments EPC-mediated angiogenesis. This study uncovers a novel epigenetic mechanism underlying this axis: RIPC-induced ZNF667 suppresses VHL transcription by competitively inhibiting the recruitment of histone-modifying enzymes MLL3/4, SETD1A, and EP300. This leads to VHL downregulation, which in turn stabilizes HIF-1α, upregulates SDF1 expression, enhances EPC recruitment and function, and ultimately improves angiogenesis and flap survival. The mechanism diagram is shown in Fig. 8. These insights not only advance our understanding of RIP’s protective mechanisms but also highlight the therapeutic potential of targeting this axis to improve outcomes in flap transplantation and other conditions involving ischemia–reperfusion injury.

## Supplementary Information

Below is the link to the electronic supplementary material.

Supplementary file 1 (TIFF 254 kb)
**RIPC mobilizes endogenous EPCs into the circulation and promotes their homing to the flap tissue**. **A**. Flow cytometry analysis of peripheral blood samples collected prior to any transfusion of labelled EPCs. The population of circulating endogenous EPCs was defined as cells positive for both CD34 and CD133. I/R injury increased the number of circulating CD34+/CD133+ EPCs compared to the sham group. RIPC pretreatment before I/R (RIPC+I/R) further significantly increased the number of circulating EPCs compared to the I/R group alone.**B**. Flow cytometry quantification of endogenous EPCs homing to the flap tissue. A single-cell suspension was prepared from the entire harvested flap. I/R injury increased the number of CD34+/CD133+ EPCs within the flap compared to the sham group. RIPC pretreatment led to a further significant increase in the number of endogenous EPCs homing to the flap compared to the I/R group alone. **C**. Control experiment for in vivoimaging. No significant fluorescence signal was detected at the flap site following intravenous injection of free DiR dye (i.e., not bound to EPCs), confirming that the specific signals observed in experimental groups are due to the accumulation of labelled EPCs. Data are presented as mean ± SD (*p < 0.05, **p < 0.01, ***p < 0.001; n=6 rats per group)

## Data Availability

The data in this study can be obtained from the corresponding author.

## References

[1] Ooms M, Winnand P, Heitzer M, Vohl N, Katz MS, Bickenbach J, et al. Blood pressure and microvascular free flap perfusion in head and neck reconstruction- a retrospective analysis. Oral Maxillofac Surg. 2025;29:85. 40407941 10.1007/s10006-025-01378-8 PMC12102101

[2] Yang T, Tan Z, Chen X, Wang F, Tao R, Tong Y, et al. Skin stretching techniques: a review of clinical application in wound repair. Plast Reconstr Surg. 2024;12:e6405. 10.1097/GOX.0000000000006405 PMC11661721 39712379

[3] Stephens CJ, Spector JA, Butcher JT. Biofabrication of thick vascularized neo-pedicle flaps for reconstructive surgery. Transl Res. 2019;211:84–122. 31170376 10.1016/j.trsl.2019.05.003 PMC6702068

[4] Tavelli L, Barootchi S, Rodriguez MV, Sugai J, Wu DT, Yu N, et al. Characterization of oral biomarkers during early healing at augmented dental implant sites. J Periodontal Res. 2025;60:206–14. 39090529 10.1111/jre.13328 PMC12024631

[5] Ersahin S, Tan Baser N, Karagoz N, Simsek G. The effect of chrysin on ischemia-reperfusion injury in the rat epigastric artery skin island flap. Ulus Travma Acil Cerrahi Derg. 2025;31:505–15. 40511759 10.14744/tjtes.2025.87041 PMC12183476

[6] Jiang S, Ma F, Lou J, Li J, Shang X, Li Y, et al. Naringenin reduces oxidative stress and necroptosis, apoptosis, and pyroptosis in random-pattern skin flaps by enhancing autophagy. Eur J Pharmacol. 2024;970:176455. 38423240 10.1016/j.ejphar.2024.176455

[7] Zhou B, Luo X. Clinical studies on the application of concentrated growth factors for enhancing the recovery from flap ischemia-reperfusion injuries. J Cosmet Dermatol. 2025;24:e70203. 40265488 10.1111/jocd.70203 PMC12015795

[8] Chen Z, Wu H, Yang J, Li B, Ding J, Cheng S, et al. Activating Parkin-dependent mitophagy alleviates oxidative stress, apoptosis, and promotes random-pattern skin flaps survival. Commun Biol. 2022;5:616. 35732814 10.1038/s42003-022-03556-w PMC9217959

[9] Freitas FA, Piccinato CE, Cherri J, Marchesan WG. Effects of pentoxyfilline and heparin on reperfusion injury island skin flaps in rats exposed to tobacco. J Surg Res. 2010;164:139–45. 20739032 10.1016/j.jss.2010.05.028

[10] Chen Z, Liu H, Li Y, Zhou Z, Qiu J, Tang Y, et al. ZNF667 attenuates leukocyte-endothelial adhesion via downregulation of P-selectin in skin flap following remote limb ischemic preconditioning. Cell Biol Int. 2021;45:1477–86. 33710682 10.1002/cbin.11586

[11] Pak CS, Moon SY, Lee YE, Kang HJ. Therapeutic effects against tissue necrosis of remote ischemic preconditioning combined with human adipose-derived stem cells in random-pattern skin flap rat models. J Invest Surg. 2021;34:1304–11. 32691637 10.1080/08941939.2020.1795750

[12] Chen Y, Yan J, Wang K, Zhu Z. Comparing the protective effects of local and remote ischemic preconditioning against ischemia-reperfusion injury in hepatectomy: a systematic review and network meta-analysis. Transl Gastroenterol Hepatol. 2024;9:13. 38716220 10.21037/tgh-23-95 PMC11074492

[13] Keskin D, Unlu RE, Orhan E, Erkilinç G, Bogdaycioglu N, Yilmaz FM. Effects of remote ischemic conditioning methods on ischemia-reperfusion injury in muscle flaps: an experimental study in rats. Arch Plast Surg. 2017;44:384–9. 28946719 10.5999/aps.2017.44.5.384 PMC5621827

[14] Guo L, Chen Y, Feng X, Sun D, Sun J, Mou S, et al. Oxidative stress-induced endothelial cells-derived exosomes accelerate skin flap survival through Lnc NEAT1-mediated promotion of endothelial progenitor cell function. Stem Cell Res Ther. 2022;13:325. 35850692 10.1186/s13287-022-03013-9 PMC9290268

[15] Jin Z, Yao C, Poonit K, Han T, Li S, Huang Z, et al. Allogenic endothelial progenitor cell transplantation increases flap survival through an upregulation of eNOs and VEGF on venous flap survival in rabbits. J Plast Reconstr Aesthet Surg. 2019;72:581–9. 30661915 10.1016/j.bjps.2018.12.042

[16] Tu TC, Nagano M, Yamashita T, Hamada H, Ohneda K, Kimura K, et al. A chemokine receptor, CXCR4, which is regulated by hypoxia-inducible factor 2alpha, is crucial for functional endothelial progenitor cells migration to ischemic tissue and wound repair. Stem Cells Dev. 2016;25:266–76. 26620723 10.1089/scd.2015.0290 PMC4742989

[17] Qin L, Xu Y, Xu Y, Ma G, Liao L, Wu Y, et al. NCOA1 promotes angiogenesis in breast tumors by simultaneously enhancing both HIF1alpha- and AP-1-mediated VEGFa transcription. Oncotarget. 2015;6:23890–904. 26287601 10.18632/oncotarget.4341 PMC4695159

[18] Hostler AC, Hahn WW, Hu MS, Rennert R, Fischer KS, Barrera JA, et al. Endothelial-specific CXCL12 regulates neovascularization during tissue repair and tumor progression. FASEB J. 2024;38:e70210. 39698751 10.1096/fj.202401307R PMC13051727

[19] Liu X, Wang J, Xu X, Zhu H, Man K, Zhang J. SDF-1 functionalized hydrogel microcarriers for skin flap repair. ACS Biomater Sci Eng. 2022;8:3576–88. 35899941 10.1021/acsbiomaterials.2c00755

[20] Chen Y, Yin W, Liu Z, Lu G, Zhang X, Yang J, et al. Exosomes derived from fibroblasts enhance skin wound angiogenesis by regulating HIF-1alpha/VEGF/VEGFR pathway. Burns Trauma. 2025 13: tkae071. 10.1093/burnst/tkae071 PMC12107542 40433567

[21] Jeon S, Cho S, Yoo S, Lee Y, Goo J, Jeong YJ, et al. Controlled delivery of HIF-1alpha via extracellular vesicles with collagen-binding activity for enhanced wound healing. J Control Release. 2025;380:330–47. 39921033 10.1016/j.jconrel.2025.02.010

[22] Wang W, Shang W, Zou J, Liu K, Liu M, Qiu X, et al. ZNF667 facilitates angiogenesis after myocardial ischemia through transcriptional regulation of VASH1 and Wnt signaling pathway. Int J Mol Med. 2022;50:129. 36043524 10.3892/ijmm.2022.5185 PMC9448299

[23] Li YZ, Chao R, Qu SL, Huang L, Zhang C. ZNF667 suppressed LPS-induced macrophages inflammation through mTOR-dependent aerobic glycolysis regulation. Curr Pharm Des. 2023;29:1361–9. 37259213 10.2174/1381612829666230530143129

[24] Dhar SS, Brown C, Rizvi A, Reed L, Kotla S, Zod C, et al. Heterozygous Kmt2d loss diminishes enhancers to render medulloblastoma cells vulnerable to combinatory inhibition of LSD1 and OXPHOS. Cell Rep. 2025;44:115619. 40286267 10.1016/j.celrep.2025.115619 PMC12324069

[25] Barber AM, Kingsley NB, Peng S, Giulotto E, Bellone RR, Finno CJ, et al. Annotation of cis-regulatory-associated histone modifications in the genomes of two Thoroughbred stallions. Front Genet. 2025;16:1534461. 40084169 10.3389/fgene.2025.1534461 PMC11903428

[26] Carolina E, Kato T, Khanh VC, Moriguchi K, Yamashita T, Takeuchi K, et al. Glucocorticoid impaired the wound healing ability of endothelial progenitor cells by reducing the expression of CXCR4 in the PGE2 pathway. Front Med. 2018;5:276. 10.3389/fmed.2018.00276 PMC6173212 30324106

[27] Weng Z, Wang C, Zhang C, Xu J, Chai Y, Jia Y, et al. All-trans retinoic acid improves the viability of ischemic skin flaps in diabetic rat models. Diabetes Res Clin Pract. 2018;142:385–92. 29936250 10.1016/j.diabres.2018.06.019

[28] Heusch G, Bøtker HE, Przyklenk K, Redington A, Yellon D. Remote ischemic conditioning. J Am Coll Cardiol. 2015;65:177–95. 25593060 10.1016/j.jacc.2014.10.031 PMC4297315

