# Supplementary figures and images for: Remote Ischemic Preconditioning Enhances Skin Flap Survival via ZNF667/SDF1-Mediated Endothelial Progenitor Cells Functions for Angiogenesis
Source: Tissue Eng Regen Med. 2026 Jun 6;23(6):881–99. doi: 10.1007/s13770-026-00817-1 (PMC13415716; doi:10.1007/s13770-026-00817-1)

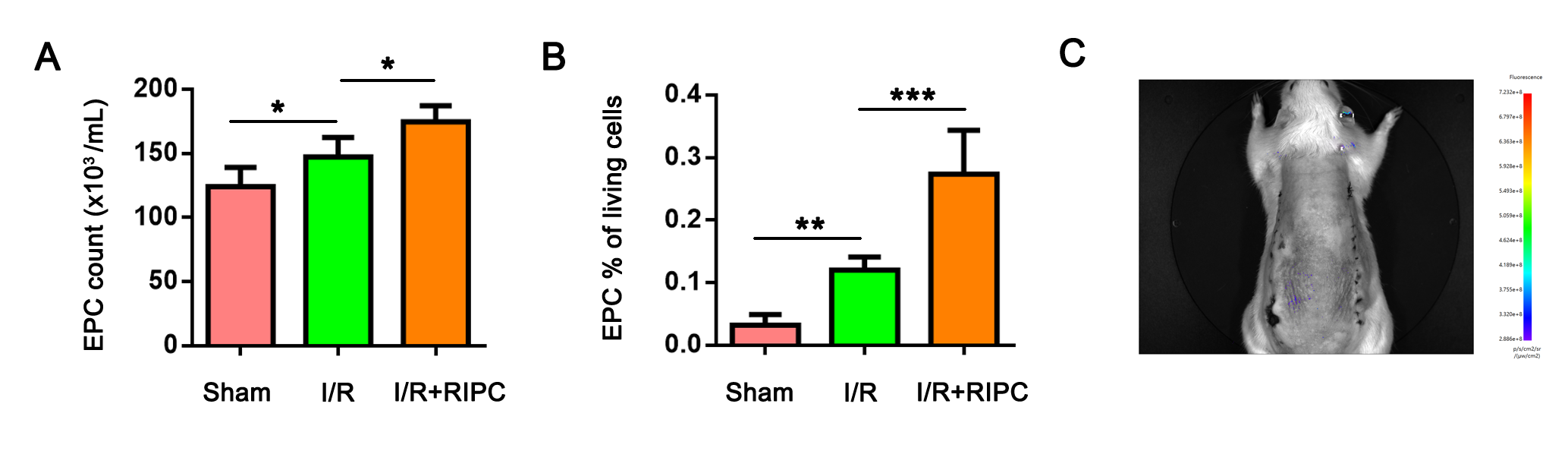

Supplement: Supplementary file 1 — (TIFF 254 kb) RIPC mobilizes endogenous EPCs into the circulation and promotes their homing to the flap tissue. A. Flow cytometry analysis of peripheral blood samples collected prior to any transfusion of labelled EPCs. The population of circulating endogenous EPCs was defined as cells positive for both CD34 and CD133. I/R injury increased the number of circulating CD34+/CD133+ EPCs compared to the sham group. RIPC pretreatment before I/R (RIPC+I/R) further significantly increased the number of circulating EPCs compared to the I/R group alone.B. Flow cytometry quantification of endogenous EPCs homing to the flap tissue. A single-cell suspension was prepared from the entire harvested flap. I/R injury increased the number of CD34+/CD133+ EPCs within the flap compared to the sham group. RIPC pretreatment led to a further significant increase in the number of endogenous EPCs homing to the flap compared to the I/R group alone. C. Control experiment for in vivoimaging. No significant fluorescence signal was detected at the flap site following intravenous injection of free DiR dye (i.e., not bound to EPCs), confirming that the specific signals observed in experimental groups are due to the accumulation of labelled EPCs. Data are presented as mean ± SD (*p < 0.05, **p < 0.01, ***p < 0.001; n=6 rats per group) [file 13770_2026_817_MOESM1_ESM.tif]
